# Assessment of Surgeon Performance of Advanced Open Surgical Skills Using a Microskills-Based Novel Curriculum

**DOI:** 10.1001/jamanetworkopen.2022.29787

**Published:** 2022-09-02

**Authors:** Anya L. Greenberg, Mohammad M. Karimzada, Riley Brian, Ava Yap, Hubert Y. Luu, Saira Ahmed, Chiung-Yu Huang, Seth A. Waits, Ryutaro Hirose, Adnan Alseidi, Joseph H. Rapp, Patricia S. O’Sullivan, Hueylan Chern, Shareef M. Syed

**Affiliations:** 1 Department of Surgery, University of California, San Francisco; 2 Department of Surgery, University of Illinois at Chicago; 3 Department of Surgery, University of Michigan, Ann Arbor

## Abstract

**Importance:**

The increase in minimally invasive surgical procedures has eroded exposure of general surgery residents to open operations. High-fidelity simulation, together with deliberate instruction, is needed for advanced open surgical skill (AOSS) development.

**Objective:**

To collect validity evidence for AOSS tools to support a shared model for instruction.

**Design, Setting, and Participants:**

This prospective cohort study included postresidency surgeons (PRSs) and second-year general surgery residents (R2s) at a single academic medical center who completed simulated tasks taught within the AOSS curriculum between June 1 and October 31, 2021.

**Exposures:**

The AOSS curriculum includes 6 fine-suture and needle handling tasks, including deep suture tying (with and without needles) and continuous suturing using the pitch-and-catch and push-push-pull techniques (both superficial and deep). Teaching and assessment are based on specific microskills using a 3-dimensional printed iliac fossa model.

**Main Outcomes and Measures:**

The PRS group was timed and scored (5-point Likert scale) on 10 repetitions of each task. Six months after receiving instruction on the AOSS tasks, the R2 group was similarly timed and scored.

**Results:**

The PRS group included 14 surgeons (11 male [79%]; 8 [57%] attending surgeons) who completed the simulation; the R2 group, 9 surgeons (5 female [55%]) who completed the simulation. Score and time variability were greater for the R2s compared with the PRSs for all tasks. The R2s scored lower and took longer on (1) deep pitch-and-catch suturing (69% of maximum points for a mean [SD] of 142.0 [31.7] seconds vs 77% for a mean [SD] of 95.9 [29.4] seconds) and deep push-push-pull suturing (63% of maximum points for a mean [SD] of 284.0 [72.9] seconds vs 85% for a mean [SD] of 141.4 [29.1] seconds) relative to the corresponding superficial tasks; (2) suture tying with a needle vs suture tying without a needle (74% of maximum points for a mean [SD] of 64.6 [19.8] seconds vs 90% for a mean [SD] of 54.4 [15.6] seconds); and (3) the deep push-push-pull vs pitch-and-catch techniques (63% of maximum points for a mean [SD] of 284.0 [72.9] seconds vs 69% of maximum points for a mean [SD] of 142.0 [31.7] seconds). For the PRS group, time was negatively associated with score for the 3 hardest tasks: superficial push-push-pull (ρ = 0.60; *P* = .02), deep pitch-and-catch (ρ = 0.73; *P* = .003), and deep push-push-pull (ρ = 0.81; *P* < .001). For the R2 group, time was negatively associated with score for the 2 easiest tasks: suture tying without a needle (ρ = 0.78; *P* = .01) and superficial pitch-and-catch (ρ = 0.79; *P* = .01).

**Conclusions and Relevance:**

The findings of this cohort study offer validity evidence for a novel AOSS curriculum; reveal differential difficulty of tasks that can be attributed to specific microskills; and suggest that position on the surgical learning curve may dictate the association between competency and speed. Together these findings suggest specific, actionable opportunities to guide instruction of AOSS, including which microskills to focus on, when individual rehearsal vs guided instruction is more appropriate, and when to focus on speed.

## Key Points

**Question:**

How do junior residents and postresidency surgeons perform on specific microskills of a novel advanced open surgical skills (AOSS) curriculum, and how might these findings guide AOSS instruction?

**Findings:**

This cohort study of 9 junior residents and 14 postresidency surgeons found validity evidence for a novel AOSS curriculum and observed differential difficulty of tasks that can be attributed to specific microskills. Findings suggest that position on the surgical learning curve may dictate the association between competency and speed.

**Meaning:**

These findings suggest specific, actionable opportunities to guide instruction of AOSS, including on which microskills to focus, when individual rehearsal vs guided instruction is appropriate, and when to focus on speed.

## Teaser

This cohort study assesses how junior general surgery residents and postresidency surgeons perform in specific microskills of an advanced open surgical skills simulation and compares performance between groups.

## Introduction

Surgical education has historically relied on an operating room–based apprenticeship whereby trainees “resided” at their hospitals to hone operative surgical skills. However, this paradigm has undergone extensive and ongoing change^1,2,3,4^ to address the evolving surgical and technological landscape. The rise in laparoscopic and robotic surgery^5^ has required trainees to develop additional, wholly different skill sets than those required for open surgery. The increase in these minimally invasive surgical techniques has been accompanied by a reciprocal decline in open procedures.^6,7,8^ Moreover, with open surgery reserved for the most complex and technically challenging cases,^9^ resident autonomy has decreased.^10^ Finally, increased administrative burden^11^ and implementation of resident duty hour restrictions^12^ have decreased resident operative experience overall.^3^ Together, these factors have eroded exposure of surgical residents to open operations and, by extension, the opportunity to master open surgical skills. Unsurprisingly, graduating surgical residents lack confidence in performing a variety of open procedures.^3^ However, open procedures still constitute a substantial proportion of surgical volume, and standard of care requires surgeons to convert from a minimally invasive to an open procedure in an emergency. Thus, surgical educators must develop curricula to enable residents to achieve mastery of open techniques by the end of training.

Broadly, the structural transformation in the surgical learning environment has forced introduction of alternative methods of instruction and preparation. Simulation centers outside the operating room are now commonplace,^13^ and the use of novel preparation techniques, such as home simulation^14^ and mental imagery,^15^ have gained traction. However, although simulation has been shown to reduce complications of clinical procedures,^16^ attention has largely been on basic surgical skills (such as suturing and knot tying needed for skin closure), bedside procedures, and the newer minimally invasive surgical techniques. High-fidelity simulators for advanced open surgical skills (AOSS) are available but can be expensive, limiting their accessibility to learners. In addition, simulation focused on operative skills performed at depth, within confined spaces, and using fine surgical instruments has been limited. Thus, in the current educational paradigm, basic open simulation models are used^17^ with the assumption that with experience, learners can intuitively apply their basic skills to overcome demanding challenges, such as patient anatomy, restricted exposure of the surgical field, or the need to be particularly gentle.

Cost-effective, high-fidelity simulators coupled with deliberate instructional design based on sound educational theory^18^ are needed to maximize learning efficiency and enhance developing technical excellence. We sought to address this gap by developing a simulator for AOSS and establishing an associated instructional infrastructure. In this study, we collect validity evidence for these tools. We specifically asked:

1. How do second-year general surgical residents (R2s) and surgeons who completed residency (ie, postresidency surgeons [PRSs]) perform on specific microskills of the AOSS simulation?
2. How do the R2s and PRSs compare with each other in performance of these skills?

In so doing, we aimed to create a shared model for instruction of AOSS.

## Methods

### Study Design and Population

This prospective cohort study of PRSs and R2s was performed at a single academic medical center. Participants completed simulated tasks taught as part of our AOSS curriculum. We followed the Strengthening the Reporting of Observational Studies in Epidemiology (STROBE) reporting guidelines. The study was declared exempt by the institutional review board of the University of California, San Francisco, which also granted a waiver of informed consent because deidentified data were used.

### AOSS Curriculum

**Figure 1.**
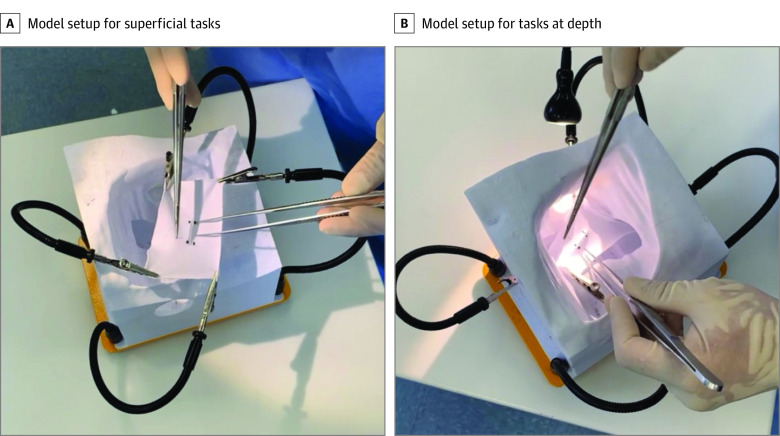
Model Setup for Advanced Open Simulation Tasks (Superficial and Deep)

The AOSS curriculum was designed for individuals in their second through fifth clinical years of residency in a surgical specialty. Participants are assumed to be proficient in basic suturing and knot tying, given their completion of the intern surgical skills curriculum, when proficiency of these tasks was evaluated. The curriculum includes 6 fine-suture (5-0 or 6-0 polypropylene [Prolene]) and needle-handling tasks using a fine needle holder and forceps. Tasks are performed on a 3-dimensional printed model of the iliac fossa (Figure 1), which recreates the depth and physical constraints of a deep vascular procedure.^19^ The model was developed using 3-dimensional Slicer open-source software, version 4.10,^20^ with a human computed tomography scan.^19^

**Figure 2.**
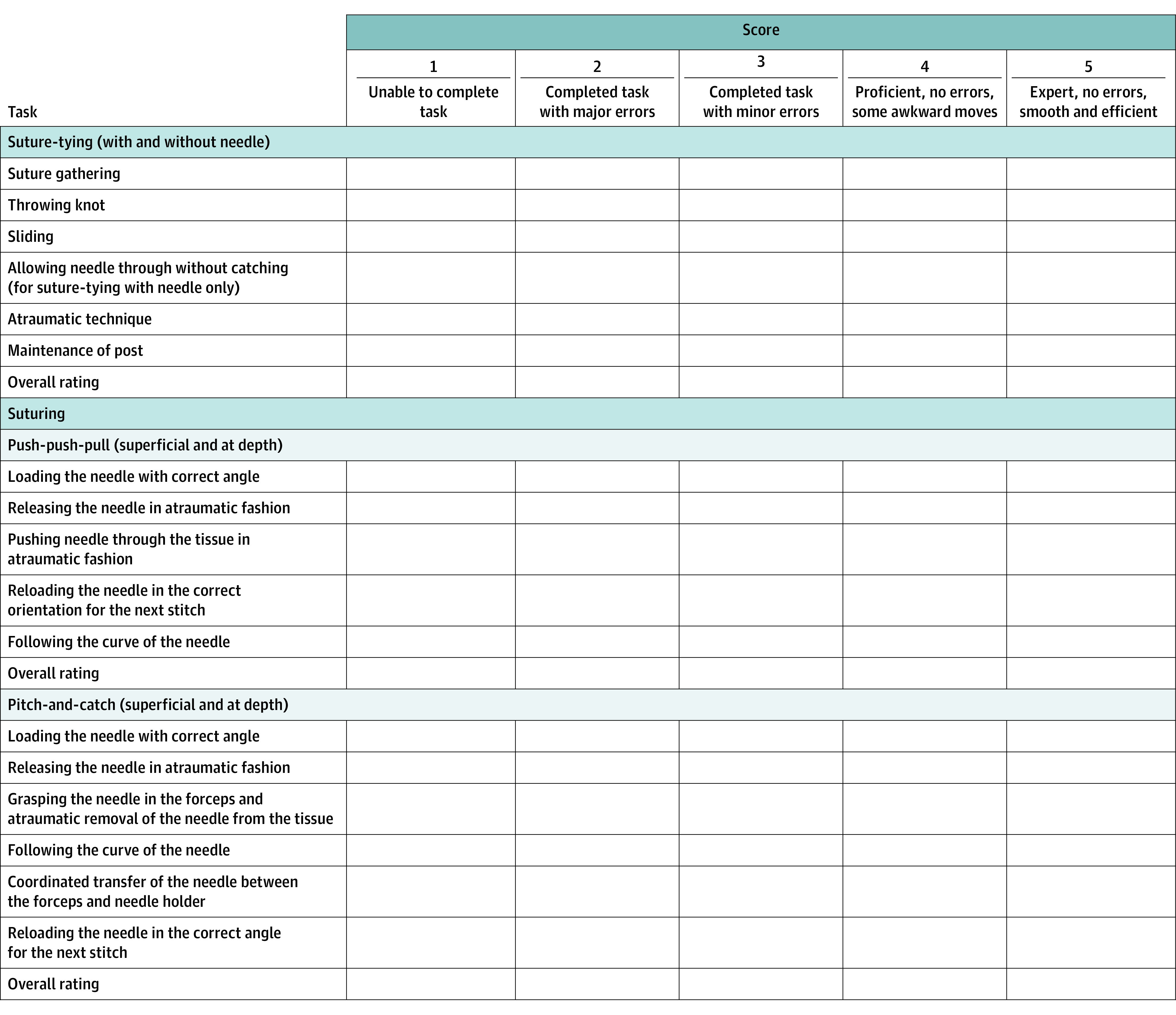
Curriculum and Grading Schema for Advanced Open Simulation Tasks

The AOSS tasks include deep suture tying (with and without needles) and continuous suturing using both superficial and deep pitch-and-catch and push-push-pull techniques (Video). The pitch-and-catch technique involves using forceps to remove the needle from the tissue and transfer it back to the needle driver. In contrast, the push-push-pull technique requires reloading the needle while it is still within the tissue without using forceps. This technique is valuable when forceps are being used for retraction or tissue stabilization during fine suturing. Although often used interchangeably during an operation, these 2 techniques require inherently different skills and thus were assessed separately. Teaching and assessment of each task is based on a specific set of psychomotor microskills (Figure 2).

**Video.** Demonstrations of the Pitch-and-Catch and Push-Push-Pull Suturing Techniques This video includes demonstrations of the pitch-and-catch and push-push-pull suturing techniques. Though often used interchangeably during an operation, these 2 techniques use different approaches to reload the needle and require inherently different skills.

### Data Collection

The PRS group, including faculty and clinical fellows, were identified by the senior author (S.M.S.). They were recruited from surgical oncology, abdominal transplantation, vascular surgery, and cardiothoracic surgery, all surgical fields in which sewing vascular anastomoses at depth using fine suturing is within the scope of practice. The PRS group did not receive compensation for their participation. The PRSs were assessed individually and timed and scored on performing 10 repetitions of each task. Scores were based on the AOSS curriculum grading schema, whereby each microskill of a given task was rated on a 5-point Likert scale, with 1 indicating unable to complete task and 5 indicating expert, no errors, smooth, and efficient (Figure 2). The total score for each task is the sum of scores for each microskill.

Six months after undergoing instruction on the AOSS tasks as part of our formal surgical skills laboratory curriculum, all R2s at our institution were invited to participate in a structured simulation session. During the session, residents rotated through a circuit of the 6 tasks. A surgical faculty rater was assigned to each station. Faculty raters measured completion time of 10 repetitions of each task and scored performance based on the same grading schema (Figure 2). Because a focus of our study was to compare 2 groups of surgeons at different points along the surgical learning curve (R2s and PRSs), examining differences by demographic factors such as race and ethnicity was outside our scope.

### Data Analysis

Scores (ie, total points for each task) and times (ie, seconds needed to perform each task) were examined descriptively using means and SDs, coefficients of variation (defined as the ratio of the SD to the mean), and IQRs. Association between time and score for each task was evaluated using Pearson correlation coefficients (ρ value). Performances by the PRS and R2 groups were compared using 2-sample *t* tests. Hypothesis tests were 2 sided with a significance threshold of *P* = .05. Statistical analysis was performed using R, version 4.1.0 (R Core Team).

## Results

### PRS Performance

Fourteen surgeons in the PRS group (3 women [21%] and 11 men [79%]; 8 attending surgeons [57%]) completed the simulation (Table 1). All 6 tasks had a mean percentage of maximum points higher than 90% (Table 2). Suture tying without the needle had the lowest mean time (36.9 [13.6] seconds), whereas the deep push-push-pull task had the highest mean time (126.8 [34.2] seconds).

**Table 1.** Characteristics of Study Participants

Characteristic	Participants, No. (%)^a^
**Postresidency surgeons**	
Gender	
Female	3 (21)
Male	11 (79)
Other	0
Role	
Attending surgeon	8 (57)
Clinical fellow	6 (43)
Specialty	
Vascular surgery	3 (21)
Transplant surgery	7 (50)
Surgical oncology	2 (14)
Cardiothoracic surgery	2 (14)
**Second-year general surgery residents**	
Gender	
Female	5 (55)
Male	4 (44)
Other	0
Residency type	
General surgery categorical	7 (78)
General surgery preliminary	1 (11)
Integrated vascular	1 (11)

^a^ Percentages may not total 100 because of rounding.

**Table 2.** Summary of PRS and R2 Performance

Performance	Score			Time, s			Association between score and time by group			
	PRS group (n = 14)	R2 group (n = 9)	*P* value	PRS group (n = 14)	R2 group (n = 9)	*P* value	PRS (n = 14)		R2 (n = 9)	
							Pearson correlation coefficient, ρ	*P* value	Pearson correlation coefficient, ρ	*P* value
**Suture tying without needle**										
Mean (SD)	28.6 (1.5)	27.0 (2.0)	.04	36.9 (13.6)	54.4 (15.6)	.01	0.17	.57	0.78	.01
Percentage of maximum 30 points	95	90		NA	NA					
Coefficient of variation^a^	0.05	0.07		0.37	0.29					
Median (IQR) [range]	29.0 (2.2) [26-30]	27.0 (2.0) [23-30]		34.0 (7.5) [22-77]	54.0 (23.0) [33-77]					
**Pitch-and-catch task (superficial)**										
Mean (SD)	33.7 (2.5)	26.9 (3.7)	<.001	75.7 (17.9)	95.9 (29.4)	.05	0.44	.12	0.79	.01
Percentage of maximum 35 points	96	77		NA	NA					
Coefficient of variation^a^	0.07	0.14		0.24	0.31					
Median (IQR) [range]	35.0 (1.0) [27-35]	27.0 (6.0) [21-32]		75.0 (15.0) [52-127]	83.0 (40.0) [50-137]					
**Suture tying with a needle**										
Mean (SD)	33.9 (1.2)	25.8 (3.8)	<.001	41.1 (11.6)	64.6 (19.8)	.002	0.06	.84	0.47	.20
Percentage of maximum 35 points	97	74		NA	NA					
Coefficient of variation^a^	0.03	0.15		0.28	0.31					
Median (IQR) [range]	34.0 (1.8) [32-35]	26.0 (5.0) [21-33]		44.0 (15.0) [21-57]	63.0 (19.0) [39-108]					
**Push-push-pull task (superficial)**										
Mean (SD)	27.6 (2.6)	25.4 (3.0)	.09	113.1 (23.3)	141.4 (29.1)	.02	0.60	.02	0.43	.25
Percentage of maximum 30 points	92	85		NA	NA					
Coefficient of variation^a^	0.09	0.12		0.21	0.21					
Median (IQR) [range]	28.5 (2.8) [21-30]	24.0 (5.0) [22-30]		109.0 (14.0) [83-159]	138.0 (46.0) [96-186]					
**Pitch-and-catch task (deep)**										
Mean (SD)	32.1 (3.2)	24.1 (4.2)	<.001	87.5 (23.3)	142.0 (31.7)	<.001	0.73	.003	0.03	.93
Percentage of maximum 35 points	92	69		NA	NA					
Coefficient of variation^a^	0.10	0.17		0.27	0.22					
Median (IQR) [range]	33.0 (4.8) [26-35]	23.0 (7.0) [20-30]		86.0 (30.8) [61-136]	146.0 (11.0) [67-188]					
**Push-push-pull task (deep)**										
Mean (SD)	28.5 (2.4)	18.8 (2.6)	<.001	126.8 (34.2)	284.0 (72.9)	<.001	0.81	<.001	0.22	.57
Percentage of maximum 30 points	95	63		NA	NA					
Coefficient of variation^a^	0.08	0.14		0.27	0.26					
Median (IQR) [range]	29.0 (1.0) [21-30]	19.0 (2.0) [14-23]		115.5 (16.8) [100-234]	290.0 (103.0) [165-400]					

Abbreviations: NA, not applicable; PRS, postresidency surgeons; R2, second-year general surgery residents.

^a^ Defined as the ratio of the SD to the mean.

The deep pitch-and-catch task had a lower mean score (32.1 [3.2] vs 33.7 [2.5]) and higher mean time (87.5 [23.3] vs 75.7 [17.9] seconds) than the same task performed superficially; the deep push-push-pull task had a higher mean score (28.5 [2.4] vs 27.6 [2.6]) and mean time (126.8 [34.2] vs 113.1 [23.3] seconds) than the same task performed superficially. Suture tying with a needle had a higher mean percentage of maximum points (97% vs 95%) and higher mean time (41.1 [11.6] vs 36.9 [13.6] seconds) than the same task without a needle. The superficial push-push-pull task had a lower mean percentage of maximum points (92% vs 96%) and higher mean time (113.1 [23.3] vs 75.7 [17.9] seconds) than the superficial pitch-and-catch task. The deep push-push-pull task had a higher mean percentage of maximum points (95% vs 92%) and higher mean time (126.8 [34.2] vs 87.5 [23.3] seconds) than the deep pitch-and-catch task.

**Figure 3.**
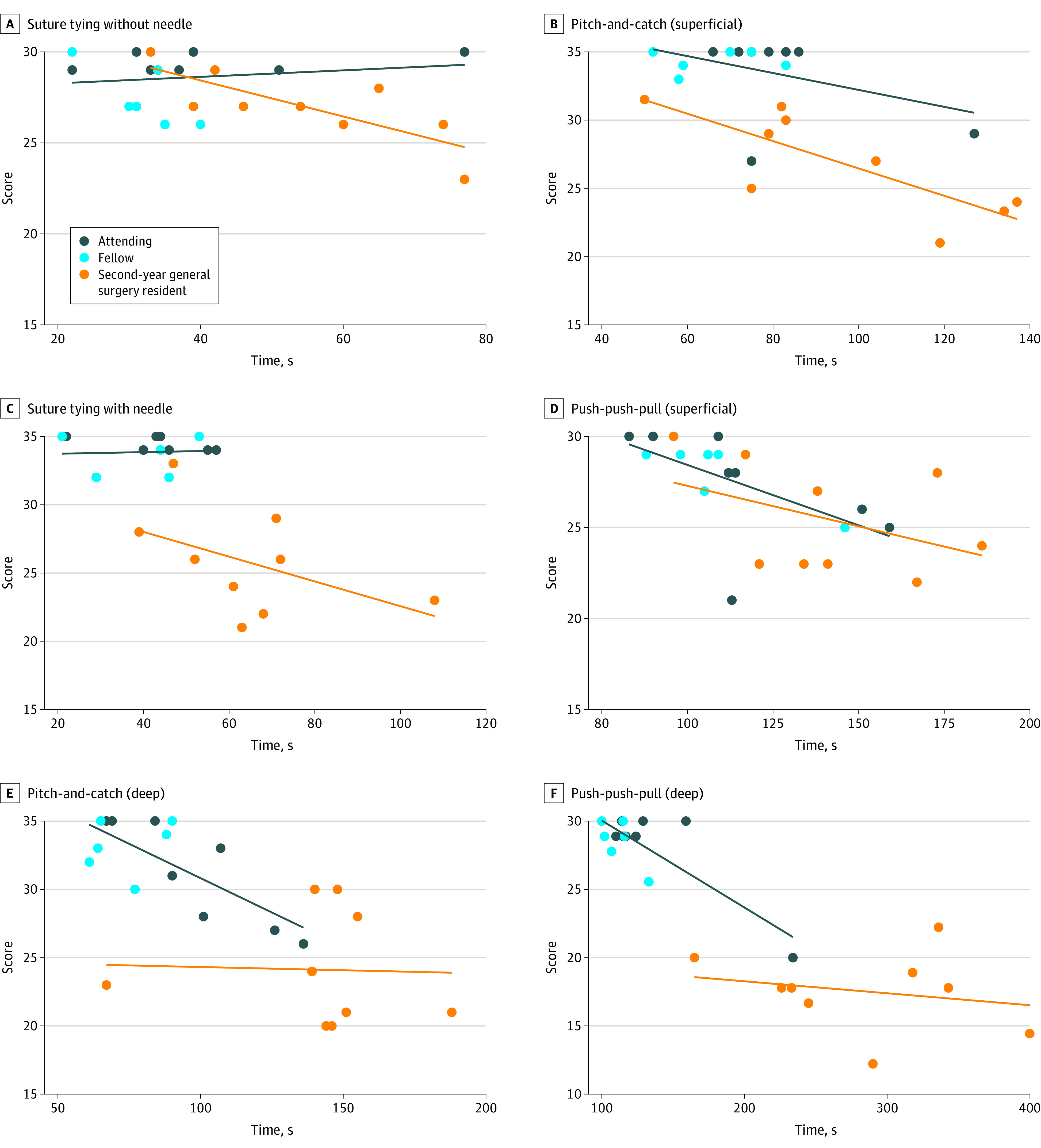
Graphic Representation of the Association Between Time and Score for Postresidency Surgeons (Attendings and Fellows) and Second-Year General Surgery Residents (R2s)

Time was negatively associated with score for the push-push-pull (superficial) (ρ = 0.60; *P* = .02), pitch-and-catch (deep) (ρ = 0.73; *P* = .003), and push-push-pull (deep) (ρ = 0.81; *P* < .001) tasks (Figure 3). Associations for other tasks did not reach statistical significance.

### R2 Performance

Nine of 10 residents in the R2 group at our institution (5 women [55%] and 4 men [44%]) completed the simulation (Table 1). Suture tying without a needle had the highest mean percentage of maximum points (90%) and lowest mean time (54.4 [15.6] seconds) (Table 2). The deep push-push-pull task had the lowest mean percentage of maximum points (63%) and highest mean time (284.0 [72.9] seconds).

Both the deep pitch-and-catch and deep push-push-pull tasks had lower mean scores (69% and 63% of maximum points, respectively) and higher mean times (142.0 [31.7] and 284.0 [72.9] seconds, respectively) than the same tasks performed superficially (77% and 85% of maximum points, respectively; and 95.9 [29.4] and 141.1 [29.1] seconds, respectively). Suture tying with a needle had lower mean percentage of maximum points (74% vs 90%) and higher mean time (64.6 [19.8] vs 54.4 [15.6] seconds) than the same task without a needle. The superficial push-push-pull task had a higher mean percentage of maximum points (85% vs 77%) and a higher mean time (113.1 [23.3] vs 75.7 [17.9] seconds) than the superficial pitch-and-catch task. The deep push-push-pull task had a lower mean percentage of maximum points (63% vs 69%) and higher mean time (284.0 [72.9] vs 142.0 [31.7] seconds) than the deep pitch-and-catch task.

Time was negatively associated with score for suture tying without a needle (ρ = 0.78; *P* = .01) and the superficial pitch-and-catch task (ρ = 0.79; *P* = .01). Associations for other tasks were not significant.

### Comparison of PRS and R2 Performance

#### Scores

Comparison of R2 and PRS scores can be seen in Table 2. Mean scores were lower for the R2 than PRS groups for all tasks; the differences for all tasks except for push-push-pull superficial reached statistical significance. The SD was higher for the R2 than PRS groups for all tasks (27.0 [2.0] vs 28.6 [1.5] for suture tying without a needle [*P* = .04]; 26.9 [3.7] vs 33.7 [2.5] for superficial pitch-and-catch [*P* < .001]; 25.8 [3.8] vs 33.9 [1.2] for suture tying with a needle [*P* < .001]; 25.4 [3.0] vs 27.6 [2.6] for superficial push-push-pull [*P* = .09]; 24.1 [4.2] vs 32.1 [3.2] for deep pitch-and-catch [*P* < .001]; and 18.8 [2.6] vs 28.5 [2.4] for deep push-push-pull [*P* < .001]). Coefficient of variation was higher for the R2 than PRS groups for all tasks (0.07 vs 0.05 for suture tying without a needle, 0.14 vs 0.07 for superficial pitch-and-catch, 0.15 vs 0.03 for suture tying with a needle, 0.12 vs 0.09 for superficial push-push-pull, 0.17 vs 0.10 for deep pitch-and-catch, and 0.14 vs 0.08 for deep push-push-pull). The IQR was higher for the R2 than PRS groups for all tasks except suture tying without a needle (26.0-28.0 vs 27.5-29.8).

#### Times

Comparison of R2 and PRS times can be seen in Table 2. Mean (SD) times were significantly higher for R2s than PRSs for all tasks (54.4 [15.6] vs 36.9 [13.6] for suture tying without a needle [*P* = .01]; 95.9 [29.4] vs 75.7 [17.9] seconds for superficial pitch-and-catch [*P* = .05]; 64.6 [19.8] vs 41.1 [11.6] seconds for suture tying with a needle [*P* = .002]; 141.4 [29.1] vs 113.1 [23.3] seconds for superficial push-push-pull [*P* = .02]; 142.0 [31.7] vs 87.5 [23.3] seconds for deep pitch-and-catch [*P* < .001]; and 284.0 [72.9] vs 126.8 [34.2] seconds for deep push-push-pull [*P* < .001]). Coefficient of variation was higher for the R2 than PRS groups for all tasks except suture tying without a needle (0.29 vs 0.37), deep pitch-and-catch (0.22 vs 0.27), and deep push-push-pull (0.26 vs 0.27). The IQR was higher for R2s than PRSs for all tasks except deep pitch-and-catch (140.0-151.0 vs 67.5-98.2).

## Discussion

This study of PRS and R2 performance using novel simulation tools revealed 3 findings that, together, offer validity evidence for the tools described herein and inform a model of AOSS instruction. First, the AOSS tasks within our curriculum are of varying levels of difficulty. Second, differential difficulty can be attributed to specific microskills. Third, the position of PRSs and R2s on the surgical learning curve may dictate the association between accuracy and speed for a given task.

Our first finding, that the AOSS tasks within our curriculum are of varying levels of difficulty, is evidenced by the spectrum of scores and times across tasks. For R2s, suture tying without a needle received the highest percentage of maximum points and took the least amount of time to complete of the tasks examined, suggesting this is the least challenging of the 6 tasks. This finding is reinforced by the relatively low variability in score. This finding is consistent with conventional surgical education, whereby surgical novices often start with knot tying as an introductory skill.^21^ In contrast, deep suturing using the push-push-pull and pitch-and-catch techniques received the lowest and second-lowest percentage of maximum points, respectively, and took the most and second-most amount of time to complete, respectively, among the tasks examined. This finding is not surprising, because operating within a restricted space and at depth requires more advanced 3-dimensional thinking, and suturing at depth alters the needle and instrument handling.^22^ Trainees find this physically constrained field particularly challenging and initially requiring unequal allocation of attention,^23^ but it is required to develop mastery applicable for clinical situations. Scores and times of remaining 3 tasks fall in between these 2 bookends and thus can be considered harder than suture tying without a needle, but easier than the 2 deep suturing tasks. Understanding task difficulty is important in establishing a goal-directed, sequential curriculum for developing proficiency in AOSS. Our findings provide preliminary objective evidence to support the relative difficulty of a select set of tasks.

Our second finding, that differential difficulty can be attributed to specific microskills, is evidenced by specific task comparisons to isolate factors driving the difference in score and time. For example, R2s had lower scores and took longer on both deep suturing tasks than their corresponding superficial tasks. This finding suggests that deep suturing is a distinct skill, independent of the individual’s ability to effectively suture. Similarly, R2s scored lower and took longer on suture tying with a needle than suture tying without a needle. This finding suggests that navigating the needle through the suture during the process of tying a knot is also a distinct skill, independent of the individual’s ability to effectively tie knots. Finally, R2s took longer on the push-push-pull than the pitch-and-catch technique superficially; they scored lower and took longer on the push-push-pull than the pitch-and-catch technique at depth. This finding suggests that the control needed to reload the needle at the appropriate angle without use of forceps in the push-push-pull technique is more challenging at depth and overall takes longer than the coordinated transfer between forceps and needle holder in the pitch-and-catch technique. These differences in difficulty are consistent with conventional thinking by practicing surgeons (ie, that tasks performed at depth are harder than those performed superficially); the present study uniquely offers data in support of this thinking.

Although this finding reiterates specific microskills that require attention, it is somewhat at odds with the current educational paradigm, which has relied on the notion that competency of a task in one setting should merely transfer over to another. Accordingly, instruction has lacked an educational basis on these advanced scenarios and has relied on the general concept of practice. The lack of exploration of curriculum alternatives and examination to optimize the instructional strategies associated with these simulations is a critical gap that our findings begin to address. Understanding these critical skills previously glossed over, together with an effective simulation model and an infrastructure for instruction and assessment, may help prepare residents for the operating room and independent practice.

Notably, our first 2 findings do not hold in 2 specific instances for PRSs. Namely, PRSs scored higher on the deep than the superficial push-push-pull task and on suture tying with a needle than without. This finding may be attributed to 3 potential reasons. First, mean scores for all tasks were more than 90% of maximum (ie, near perfect), thus limiting the potential variability at that level. Second, it is possible that increased focus was placed on the more difficult tasks by PRSs, thus resulting in slightly higher scores for the more difficult counterpart tasks. Finally, PRSs may disproportionately perform tasks at depth (relative to superficially) in their day-to-day practice and are perhaps more adept at these techniques from sheer repetition. Outside these 2 instances, the same findings can be seen in PRSs as R2s, further reinforcing the variability in difficulty in the tasks and the set of specific microskills as opportunities to guide instruction.

Our third finding, that the position of PRSs and R2s on the learning curve may dictate the association between accuracy and speed for a given task, is supported by our correlation analysis. For PRSs, a negative association between accuracy and speed is seen for the 3 most difficult tasks (both deep suturing tasks and superficial push-push-pull tasks), whereas the correlation for the least difficult tasks (both suture tying tasks and the superficial pitch-and-catch task) is weak (ie, ρ < 0.5) and not significant. Notably, for those tasks where the correlation is not significant, the variability in score is minimal, as exhibited by the coefficients of variation. This is not unexpected for PRSs, who achieved at least 95% of maximum points on these tasks. Lack of differentiation in score within this PRS group is likely the driver for the insignificant association between score and time. On the other hand, the fact that variability in score for both deep suturing tasks and the superficial push-push-pull task is greater than that of both suture tying tasks and the superficial pitch-and-catch task even among PRSs further reinforces the higher level of difficulty of these tasks.

In contrast, for R2s, variability in scores was considerable (and higher than PRS) for each task, thus allowing for adequate differentiation. A negative association between accuracy and speed was found for the 2 least difficult tasks (suture tying without a needle and the superficial pitch-and-catch technique) whereas the association for the most difficult tasks (suture tying with a needle, the superficial push-push-pull task, and both deep suturing tasks) is not significant. We posit this difference is driven by the position of R2s along the learning curve of each of these tasks. It is plausible that for the easiest tasks, where R2s are further along the learning curve, variation in score is more likely driven by an isolated awkward move or a one-off mistake that also increases time, rather than a general deficiency in competency. On the other hand, in the harder tasks for which R2s are not as far along the learning curve, variation in both score and time may be driven by a conglomerate of awkward moves or mistakes due to a general deficiency in competency of a particular task.

Together these 3 findings suggest specific, actionable opportunities to guide instruction. For easier tasks in which a group of learners has achieved competency (ie, where there is a significant association between time and score), individual rehearsal (eg, through a home simulator) to minimize mistakes and ad hoc feedback to address awkward moves may be appropriate. In contrast, for more difficult tasks in which a group of learners has not yet achieved competency (ie, where there is no association between time and score), the notion of the zone of proximal development, as expanded by Wass and Golding,^18^ offers a useful framework. The zone of proximal development represents a set of tasks that learners initially can only complete with assistance but eventually can do on their own through a learning process that effectively expands their zone of proximal development. Importantly, scaffolding to “assist learners in accomplishing tasks but also enable them to learn from the experience”^24^ is needed for independence to be reached.^25^ Moreover, a safe, supportive environment to enable engagement is suggested to influence learning.^18^ Applying this model to more advanced tasks where a group of learners have not yet achieved competency (ie, where there is not a significant association between time and score) suggests that hands-on instruction, such as within skills laboratories, may be beneficial at this stage. Further, following a set of transparent microskills (ie, the scaffold) in a low-risk setting (ie, simulation outside the operating room) may expand the zone of proximal development and facilitate learning.

Although both accuracy and efficiency are critical for practicing surgeons, at present no standard approach to teaching speed exists. The present study helps us to clarify whether a focus on speed may be appropriate. The negative association between completion time and competency for the least challenging tasks (ie, tasks at the learner’s level) suggests that eliminating one-off errors and awkward moves that take time (and worsen the outcome) may be associated with a concomitant increase in speed. Moreover, instruction of more advanced tasks for which there is a general deficiency should focus on microskills to achieve competency. Thus, our findings suggest that educators should globally concentrate on technique rather than speed.

### Limitations

Our findings should be viewed in the context of several limitations. Consequences of the erosion of surgical resident exposure to open cases are multifaceted, including not only the loss of opportunity to master specific AOSS (such as suturing and deep suture tying) but also to complete the steps of the operation, to obtain exposure, and to achieve hemorrhage control, among others. Our study deliberately focuses on instruction of AOSS as a starting point to address the overall issue. With ours as the foundation, future work should seek to address other gaps in open operative surgical education. Within this confine, our study identifies insights for instruction at large, rather than identifying remediation techniques for individual outliers. Moreover, the single-institution nature of this study, further limited by the size of our R2 class, may introduce bias. Finally, our study represents only 2 discrete groups along the learning curve of AOSS. These limitations should be addressed through a longitudinal, multicenter study that uses the simulator and standard curriculum to confirm and expand our findings. Such a setting would enable not only increasing the statistical power, but testing the same group of trainees year-over-year may indicate the relative effect of the general learning curve from the impact of the advanced curriculum. Importantly, future work should assess the impact of this curriculum on intraoperative performance. Nonetheless, our study reveals important insights that provide tangible, actionable guidance to surgical educators around the instruction of AOSS.

## Conclusions

The findings of this cohort study offer validity evidence for a novel AOSS curriculum, reveal differential difficulty of tasks that can be attributed to specific microskills, and suggest that a learner’s position on the surgical learning curve may dictate association between competency and speed. Together these findings suggest specific, actionable opportunities to guide instruction of AOSS, including on which microskills to focus, when individual rehearsal vs guided instruction is more appropriate, and when to focus on speed.
